# Molecular subtypes predict therapeutic responses and identifying and validating diagnostic signatures based on machine learning in chronic myeloid leukemia

**DOI:** 10.1186/s12935-023-02905-x

**Published:** 2023-04-06

**Authors:** Fang-Min Zhong, Fang-Yi Yao, Yu-Lin Yang, Jing Liu, Mei-Yong Li, Jun-Yao Jiang, Nan Zhang, Yan-Mei Xu, Shu-Qi Li, Ying Cheng, Shuai Xu, Bo Huang, Xiao-Zhong Wang

**Affiliations:** Jiangxi Province Key Laboratory of Laboratory Medicine, Center for Laboratory Medicine, Department of Clinical Laboratory, Jiangxi Provincial Clinical Research, The Second Affiliated Hospital of Nanchang University, No. 1 Minde Road, Nanchang, 330006 Jiangxi Provence China

**Keywords:** Chronic myeloid leukemia, Tumor microenvironment, Diagnosis, Therapeutic response, Machine learning

## Abstract

Chronic myeloid leukemia (CML) is a hematological tumor derived from hematopoietic stem cells. The aim of this study is to analyze the biological characteristics and identify the diagnostic markers of CML. We obtained the expression profiles from the Gene Expression Omnibus (GEO) database and identified 210 differentially expressed genes (DEGs) between CML and normal samples. These DEGs are mainly enriched in immune-related pathways such as Th1 and Th2 cell differentiation, primary immunodeficiency, T cell receptor signaling pathway, antigen processing and presentation pathways. Based on these DEGs, we identified two molecular subtypes using a consensus clustering algorithm. Cluster A was an immunosuppressive phenotype with reduced immune cell infiltration and significant activation of metabolism-related pathways such as reactive oxygen species, glycolysis and mTORC1; Cluster B was an immune activating phenotype with increased infiltration of CD4 + and CD8 + T cells and NK cells, and increased activation of signaling pathways such as interferon gamma (IFN-γ) response, IL6-JAK-STAT3 and inflammatory response. Drug prediction results showed that patients in Cluster B had a higher therapeutic response to anti-PD-1 and anti-CTLA4 and were more sensitive to imatinib, nilotinib and dasatinib. Support Vector Machine Recursive Feature Elimination (SVM-RFE), Least Absolute Shrinkage Selection Operator (LASSO) and Random Forest (RF) algorithms identified 4 CML diagnostic genes (HDC, SMPDL3A, IRF4 and AQP3), and the risk score model constructed by these genes improved the diagnostic accuracy. We further validated the diagnostic value of the 4 genes and the risk score model in a clinical cohort, and the risk score can be used in the differential diagnosis of CML and other hematological malignancies. The risk score can also be used to identify molecular subtypes and predict response to imatinib treatment. These results reveal the characteristics of immunosuppression and metabolic reprogramming in CML patients, and the identification of molecular subtypes and biomarkers provides new ideas and insights for the clinical diagnosis and treatment.

**Supplementary Information:**

The online version contains supplementary material available at 10.1186/s12935-023-02905-x.

## Introduction

Chronic myeloid leukemia (CML) is a hematological tumor with malignant proliferation of hematopoietic stem cells [1]. The Philadelphia (Ph) chromosome formed by the translocation of chromosomes 9 and 22, namely t(9;22), is closely related to disease, and the recombined Breakpoint cluster region-Abelson (BCR-ABL1) fusion gene encodes an oncoprotein with strong tyrosine kinase activity that is a key factor contributing to CML pathogenesis. CML is divided into chronic phase (CP), accelerated phase (AP) and blast phase (BP) [2]. The main therapeutic drugs for CML are tyrosine kinase inhibitors (TKIs), among which imatinib (IM) is widely used as a first-generation TKI with good therapeutic benefits [3]. However, over 5 years of follow-up, approximately 1 in 5 CML patients developed resistance to IM [4], which included primary resistance and relapse after treatment response. For the former, the BCR-ABL1 kinase has an independent mechanism; for the latter, it is mainly due to mutations in the structural domain of the BCR-ABL1 kinase [5]. Moreover, the long-term use of TKI is associated with many complications that affect patients' quality of life [6]. Therefore, it is important to explore the pathogenesis of CML in depth and identify new diagnostic biomarkers or therapeutic targets.

With the development of next-generation sequencing (NGS) technologies, studies based on the levels of gene expression and regulation have shed light on the pathogenesis and malignant phenotypes of many diseases [7–10]. The incorporation of NGS into the diagnosis, risk stratification and therapeutic evaluation of CML patients is of great importance [11]. Additional novel biomarkers can be identified by systematically analyzing the differences in gene expression profiles between CML samples and normal samples [12, 13]. However, most of the current studies related to CML are limited to a single molecule or signal pathway, lacking large samples and multidimensional exploration. Comprehensive analysis of gene expression characteristics, signaling pathway activities and immune cell infiltration levels in CML samples may contribute to our understanding of CML pathogenesis and tumor microenvironment (TME).

In this study, we focused on multiple CML transcriptome sequencing cohorts, and found more downregulated genes and significantly fewer immune cells in CML samples compared to normal samples. CML patients can be further classified into immune activating and immunosuppressive phenotypes and have different predictive responsiveness to treatment with TKI drugs and immune checkpoint inhibitors. We further identified 4 genes with diagnostic value by multiple machine learning methods and validated them in validation cohorts.

## Methods

### Data acquisition and processing

The datasets GSE13159, GSE144119, GSE100026 were downloaded from the Gene Expression Omnibus (GEO) database. For GSE13159 (76 CML samples and 74 normal samples), we downloaded the original “cel” files and normalized it using the robust multiarray averaging (RMA) method. For GSE144119 (16 CML samples and 11 remission samples, as well as 6 normal samples), we downloaded the count data and converted it to transcripts per kilobase million (TPM) values. GSE100026 (10 CML samples and 5 normal samples) is our own CML data, and we converted the fragments per kilobase of transcript per million (FPKM) values to TPM values for subsequent validation. In this study, GSE13159 was used as the discovery cohort for systematic analysis, GSE144119 and GSE100026 were used as the validation cohorts. In addition, GSE13159 contained samples from 750 patients with acute lymphoblastic leukemia, 542 patients with acute myeloid leukemia, 448 patients with chronic lymphocytic leukemia, and 206 patients with myelodysplastic syndromes, which were further used in the differential diagnosis of CML.

### Identification of differentially expressed genes (DEGs)

We performed differential expression analysis using the R package “limma” to identify DEGs between CML and normal samples in the dataset. The threshold for DEGs was set at an absolute value of log2 fold change |log2FC|> 1 and an adjusted P value < 0.05.

### Functional enrichment analysis and construction of protein–protein interaction (PPI) network

Functional enrichment analysis was performed by the R package “clusterProfiler”. Kyoto Encyclopedia of Genes and Genomes (KEGG) and Gene Ontology (GO) analyses were used for the identification of biological functions associated with DEGs; Gene Set Enrichment Analysis (GSEA) and Gene Set Variation Analysis (GSVA) algorithms quantified the activity of corresponding signaling pathways or biological behaviors by calculating the enrichment scores of gene sets, and the GSVA score was calculated based on the overall position of the gene set genes in the expression ranking of all genes, and the higher the overall expression level of these genes, the higher the GSVA score. The STRING website (https://cn.string-db.org/) was used to construct PPI networks, and Cytoscape software was further used to calculate and identify sub networks with the highest connectivity.

### Identification of molecular subtypes based on DEGs

We performed consensus clustering analysis on the expression of DEGs based on the “consensusclusterplus” package to identify the molecular subtypes of CML, and performed 1000 iterations to ensure the reliability and stability of classification. Principal component analysis (PCA) was used to validate the classification.

### Estimation of immune cell infiltration

CIBERSORT is a deconvolution algorithm that quantifies the proportion of immune cell infiltration in a sample (22 different cell types) based on gene expression profiles. We further compare the immune cell infiltration characteristics in CML samples and normal samples to reveal differences in the immune microenvironment between the two.

### Construction of upstream regulatory network

We used the eXpression2Kinases website (X2K) (https://amp.pharm.mssm.edu/X2K/) for the construction of upstream regulatory network, where regulatory correlations between transcription factors (TFs), kinases and intermediate proteins were calculated based on hypergeometric P values.

### Prediction of treatment response for different molecular subtypes

The half-maximal inhibitory concentrations (IC50) of different patient samples to therapeutic drugs were predicted based on drug response data of blood cell lines from the Cancer Genome Project (CGP) database (https://cancer.sanger.ac.uk/cosmic) via the "pRRophetic" package. Tumor Immune Dysfunction and Exclusion (TIDE, http://tide.dfci.harvard.edu/) was considered a good predictor of immunotherapeutic response for molecular subtypes [14, 15]. The SubMap algorithm (https://cloud.genepattern.org/gp) was used to predict immunotherapy response to anti-PD-1 and anti-CTLA4 immune checkpoint inhibitors across different molecular subtypes.

### Identification of diagnostic biomarkers for CML

We use three machine learning algorithms [16], Support Vector Machine Recursive Feature Elimination (SVM-RFE), Least Absolute Shrinkage Selection Operator (LASSO) and Random Forest (RF), to filter variables significantly associated with CML. SVM-RFE is based on the support vector machine algorithm to rank the variable attributes and further identify the best variables using the “e1071” package. “LASSO” was performed using the “glmnet” package as a regression analysis algorithm that applies regularization to variable selection. RF identifies the categorical importance of variables based on decision tree theory. We applied three algorithms to identify overlapping genes as diagnostic biomarkers of CML in the GSE13159 dataset and validated them in GSE144119 and GSE100026 cohorts. To further improve the diagnostic value of the biomarkers, we calculated the regression coefficients of overlapping genes by LASSO regression analysis and constructed a risk-score diagnostic model of CML based on the following formula:

$$Risk\,score = \sum\nolimits_{1}^{i} {(Coefi*ExpGenei)} ,$$

where i is the diagnostic model gene, “Coef” and “ExpGene” are the non-0 regression coefficient and the expression value of it.

### Construction of a miRNA regulatory network for CML diagnostic genes

We identified miRNAs with binding sites to CML diagnostic genes using the ENCORI database (https://starbase.sysu.edu.cn/) and screened for interactions of miRNA-target demonstrated by at least three predicting programs.

### RNA isolation and quantitative real-time PCR (RT-qPCR)

Our project was approved by the Ethics Committee of the Second Affiliated Hospital of Nanchang University, and peripheral blood samples from 16 CML patients and 16 normal controls were collected. Informed consent was obtained from all participants before using the samples. We extracted RNA from mononuclear cells and reverse transcribed it. TAKARA kit (Japan) was used to perform RT-PCR on the ABI7500 instrument to determine the expression of 4 diagnostic genes. The results were calculated using 2^−△△CT^ method. The primer sequences are as follows. HDC: F, ATGCTGATGAGTCCTGCCTAAAT; R, TGTCATCCACAGGCAGAAATTTC. SMPDL3A: F, CACCTCATGTTCCTGTACCTGAA; R, ACCTGTGGCCAATAGTCATGATT. IRF4: F, CAGATCGACAGCGGCAAGTA; R, TGTCGATGCCTTCTCGGAAC. AQP3: F, CCTTCTTGGGTGCTGGAATAGTT; R, GGCCAGCACACACACGATAAG. GAPDH: F, ATGGTGAAGGTCGGTGTGAA; R, ATGGTGAAGGTCGGTGTGAA.

### Statistical analysis

R software and corresponding packages were used for calculations and analysis. We used Wilcoxon rank sum test and Kruskal–Wallis test to determine differences between two or more groups. Spearman’s method used to analyze the correlation of variables. Receiver operating characteristic (ROC) curve analysis was used to determine the diagnostic value of biomarkers. A two-sided P value of < 0.05 was considered statistically significant.

## Results

**Fig. 1:**
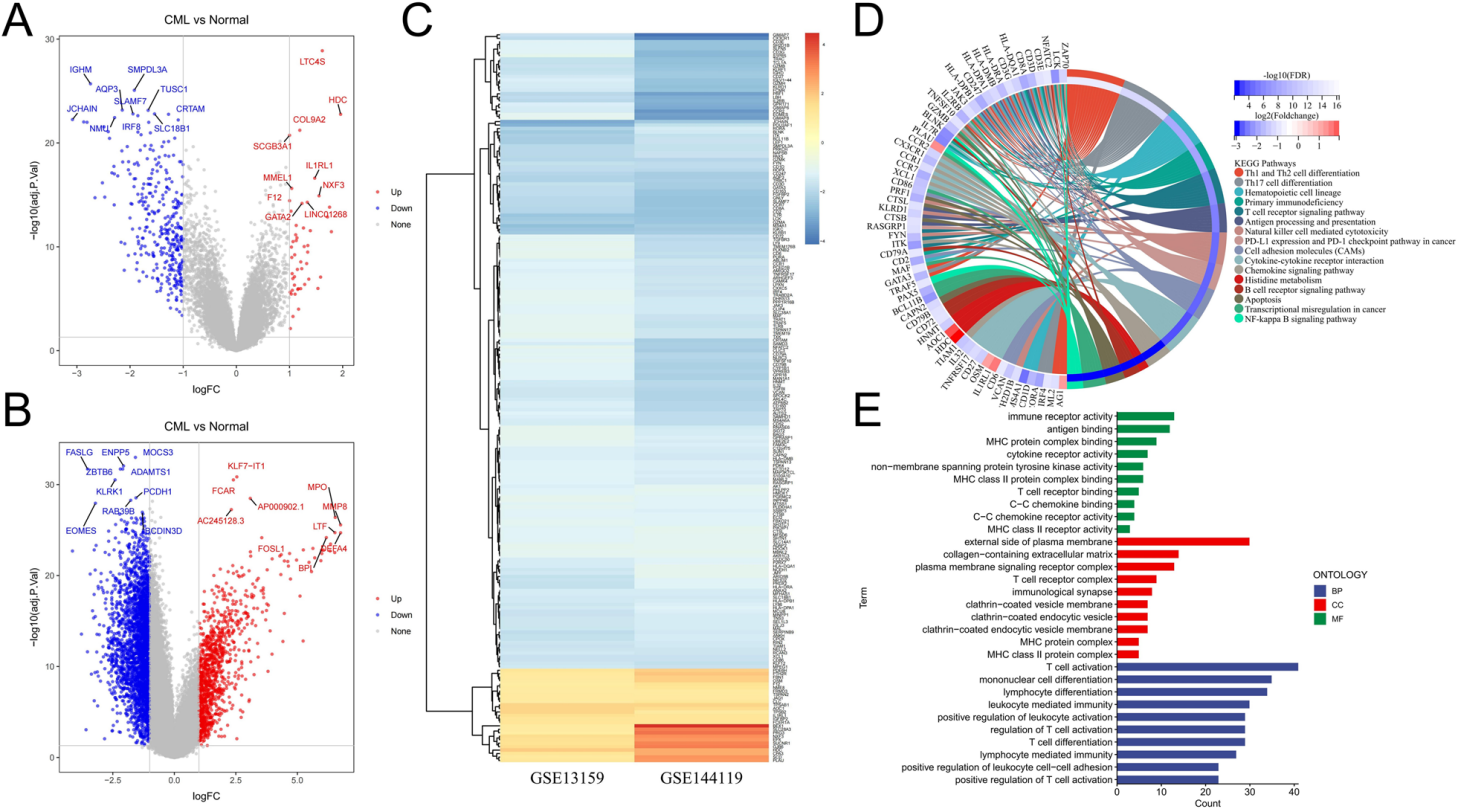
Identification of differentially expressed genes (DEGs) between CML and normal samples. **A**, **B** Volcano plots of datasets GSE13159 (**A**) and GSE144119 (**B**) from the GEO database; blue dots represent up-regulated DEGs, gray dots represent nonsignificant genes, and red dots represent down-regulated DEGs. The top 10 high- and low-expressing DEGs with the smallest adjusted P-values would be listed. **C** The heatmap shows DEGs with common expression trend in both cohorts. **D** KEGG enrichment analysis of DEGs. **E** GO annotation analysis of DEGs

To better reveal the differences in gene expression levels between CML and normal samples, we analyzed two cohorts of CML sequencing data. We identified a total of 378 DEGs in the GSE13159 cohort and 3937 in the GSE144119 cohort (Fig. 1A, B), for a total of 210 DEGs with the same expression trend in both cohorts (Fig. 1C), and these genes were used for subsequent analysis. We observed in both cohorts that fewer genes were up-regulated in expression and more genes were down-regulated in expression in CML samples compared to normal samples. We further performed functional analysis of these shared DEGs, and the results of KEGG analysis showed that most of the down-regulated genes were mainly enriched in immune-related signaling pathways, such as Th1 and Th2 cell differentiation, primary immunodeficiency, T cell receptor signaling pathway, antigen processing and presentation, natural killer cell mediated cytotoxicity, PD-L1 expression and PD-1 checkpoint pathway in cancer, cytokine-cytokine receptor interaction, chemokine signaling pathway, B cell receptor signaling pathway (Fig. 1D). GO annotations also indicated that the molecular functions of these genes and the biological processes involved are focused on inflammatory and immune signatures (Fig. 1E).

### Immune cell infiltration and the upstream regulatory network of DEGs

**Fig. 2:**
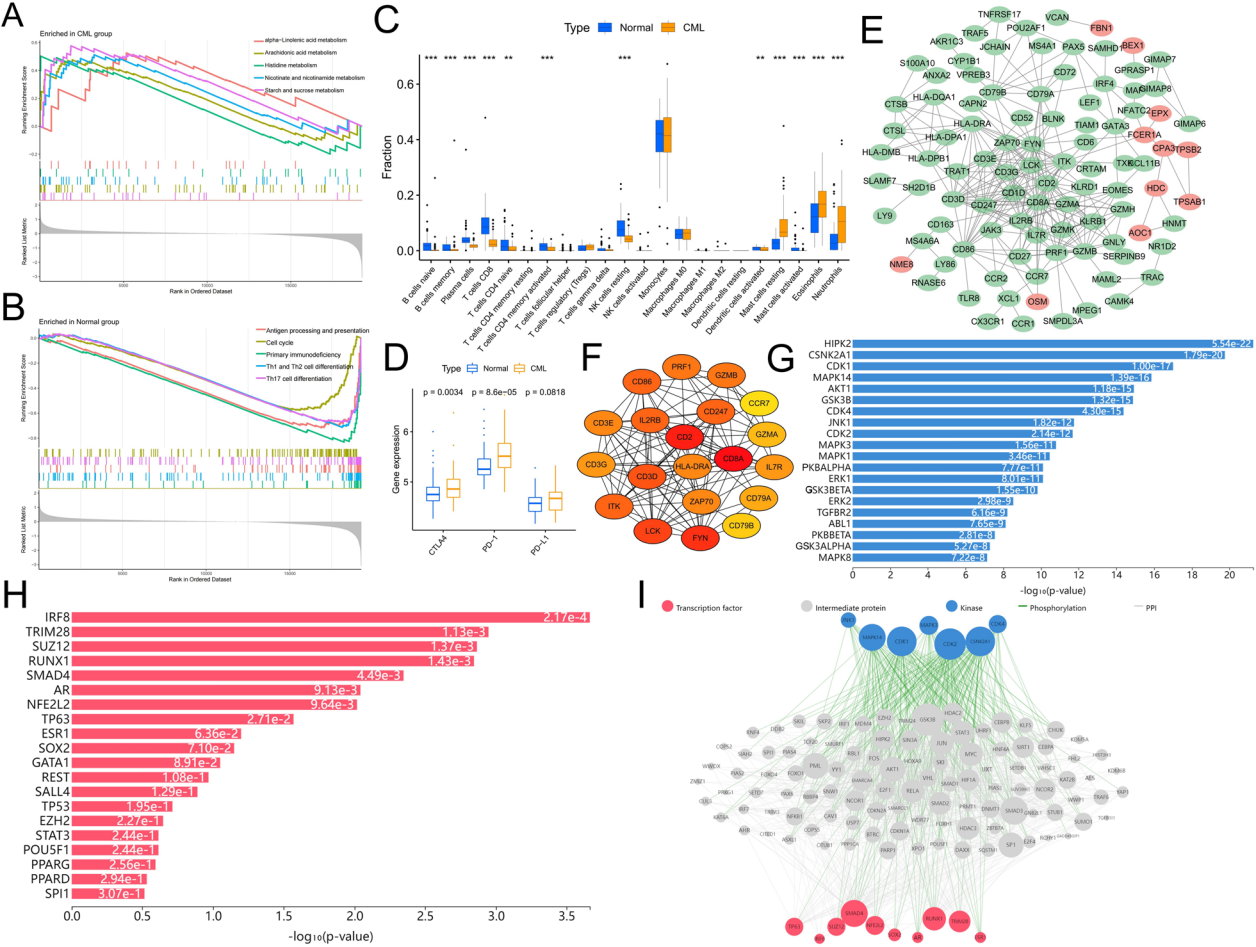
Analysis of immune cell infiltration and prediction of upstream regulatory network according to the DEGs. **A** GSEA analysis of enrichment pathways in the CML group. **B** GSEA analysis of enrichment pathways in the Normal group. **C** Differences in infiltration of 22 immune cells between CML and normal samples. **D** Differences in expression of immune checkpoints between CML and normal samples. **E** PPI network of the DEGs. **F** The subnetwork of the top 20 most connected genes in the PPI network. **G** Kinases and **H** transcription factors according to the predictions of the DEGs. **I** Regulatory network diagram according to the prediction of the DEGs. Nodes’ size is scaled proportional to the corresponding degree

We further explored the differences in biological characteristics between CML and normal samples. the results of GSEA enrichment analysis reconfirmed that the activity of immune-related signaling pathways was significantly reduced in CML, but that metabolic-related pathways such as alpha-linolenic acid metabolism, arachidonic acid metabolism, histidine metabolism, nicotinate and nicotinamide metabolism, and starch and sucrose metabolism were significantly activated (Fig. 2A, B). Immune cell infiltration analysis showed that naive and memory B cells, plasma cells, CD4 + and CD8 + T cells, resting NK cells, activated dendritic cells and mast cells were significantly reduced in CML samples compared to normal samples, while eosinophils and resting mast cells were significantly enriched in CML samples, except for neutrophils (Fig. 2C). All of our data indicated that the CML samples were in an immunosuppressed state. Subsequently, we explored the expression of immune checkpoint genes between the two groups. Increased expression of PD-L1, PD-1, and CTLA4 was observed in the CML samples (Fig. 2D). These results suggest that CML patients exhibit inertia in anti-tumor immunity, which may be an important contributor to the progression of CML.

DEGs may be important factors involved in abnormal alterations of the biological features of CML. We performed PPI network construction for these DEGs, in which the DEGs with downregulated expression were located in the core of the network and those with upregulated expression were located at the edges (Fig. 2E). We extracted the top 20 genes with the highest connectivity, and these genes such as CD8A, CD3D, CD3E, CD3G, GZMA, GZMB, CD86, ITK, CD79A, CD79B are closely related to the function of immune cells (Fig. 2F). We further used DEGs to predict the upstream regulatory network of CML pathogenesis, which includes TFs, kinases and intermediate proteins. The most significantly associated kinases included HIPK2, CSNK2A1, CDK1, MAPK14, AKT1, GSK3B, CDK4, JNK1, CDK2, MAPK3, and MAPK1 (Fig. 2G). The most significantly associated TFs included IRF8, TRIM28, SUZ12, RUNX1, SMAD4, AR, NFE2L2, TP63, ESR1, SOX2 (Fig. 2H). A total of 105 intermediate proteins with 1437 edges were linked to the TFs and kinases (Fig. 2I).

### Distinct molecular subtypes in CML

**Fig. 3:**
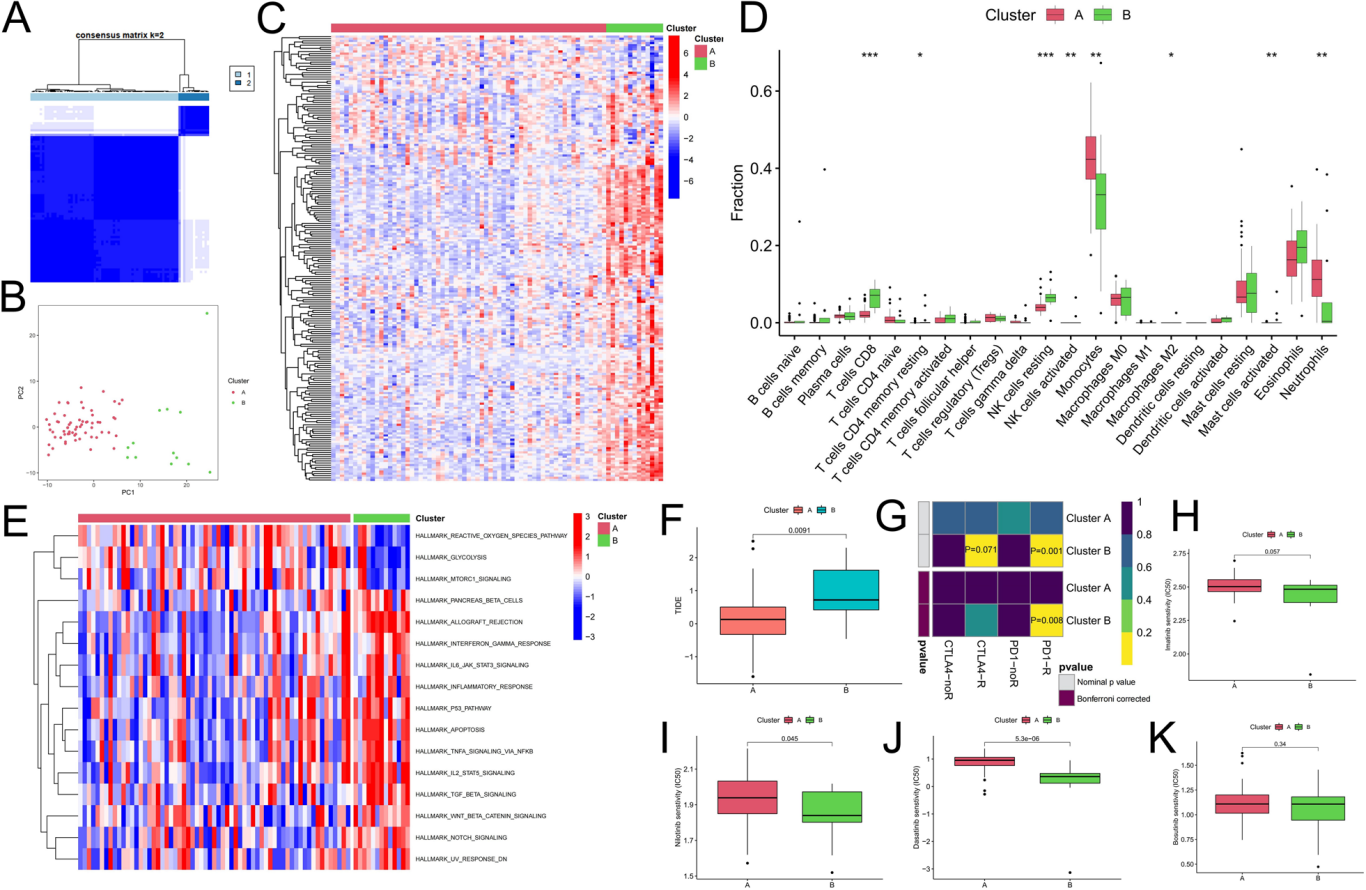
Identification of molecular subtypes of CML and prediction of drug response in different subtypes. **A** The consensus clustering algorithm divided CML patients into two different molecular subtypes based on the expression of DEGs. **B** PCA algorithm was used to verify the classification reliability of the two molecular subtypes. **C**–**F** Differences in expression of DEGs (**C**), infiltration of 22 immune cells (**D**), activity of tumor hallmark gene sets (**E**) and TIDE scores (**F**) between the two molecular subtypes. (**G**) Differences in the therapeutic response of the two molecular subtypes to immune checkpoint inhibitors. **H**–**K** Differences in therapeutic sensitivity of the two molecular subtypes to four TKIs

We performed consensus clustering of CML patients based on the expression of DEGs and identified two molecular subtypes (Cluster A and Cluster B) (Fig. 3A). The PCA algorithm further validated the reliability of the classification (Fig. 3B). Compared to Cluster A, most DEGs were upregulated in Cluster B (Fig. 3C). Moreover, immune infiltration analysis showed that CD8 + T cells, resting CD4 + memory T cells, resting and activated NK cells were significantly enriched in Cluster B, and monocytes and neutrophils were infiltrated at higher levels in Cluster A (Fig. 3D). In the GSE144119 cohort, we used the same approach to identify two molecular subtypes with familiar expression and immune infiltration characteristics (Additional file 1: Figure S1A-S1B). Meanwhile, we used the GSVA algorithm to compare the difference in activity of the tumor hallmark gene set in the two molecular subtypes (Fig. 3E). We found that the reactive oxygen species (ROS) pathway, glycolytic and mTORC1 signaling pathways were more active in Cluster A; the gene sets of interferon gamma (IFN-γ) response, IL6-JAK-STAT3 signaling pathway, IL2-STAT5 signaling pathway, inflammatory response, P53 pathway, and IL2-JAK-STAT5, Notch, TGF-β, TNF-α, WNT-β signaling pathways had higher enrichment scores in Cluster B. These results suggest that the two molecular subtypes are significantly different, with Cluster B exhibiting an inflammatory and immune activating phenotype and Cluster A exhibiting immune suppression, as well as a more active energy metabolism characteristic.

### Prediction of treatment response for different molecular subtypes

We further predicted the treatment response of different molecular subtypes. The TIDE score reflects the immune escape ability of tumor cells, and we observed a higher TIDE score for Cluster B than Cluster A (Fig. 3F), suggesting that Cluster B has a higher immune escape ability, implying that patients of this subtype may benefit more from immunotherapy. We mapped the expression profiles of CML patients with another dataset containing 47 melanoma patients who responded to immunotherapy. The results showed that patients with Cluster B were more likely to respond to anti-CTLA4 and anti-PD-1 therapies (Fig. 3G). We then predicted the response of different molecular subtypes to TKIs commonly used for CML treatment, and the results showed that Cluster B patients had higher therapeutic sensitivity to imatinib, nilotinib and dasatinib (Fig. 3H, J); however, there was no significant difference in therapeutic sensitivity to bosutinib between the two groups (Fig. 3K).

### Identification and validation of diagnostic biomarkers

**Fig. 4:**
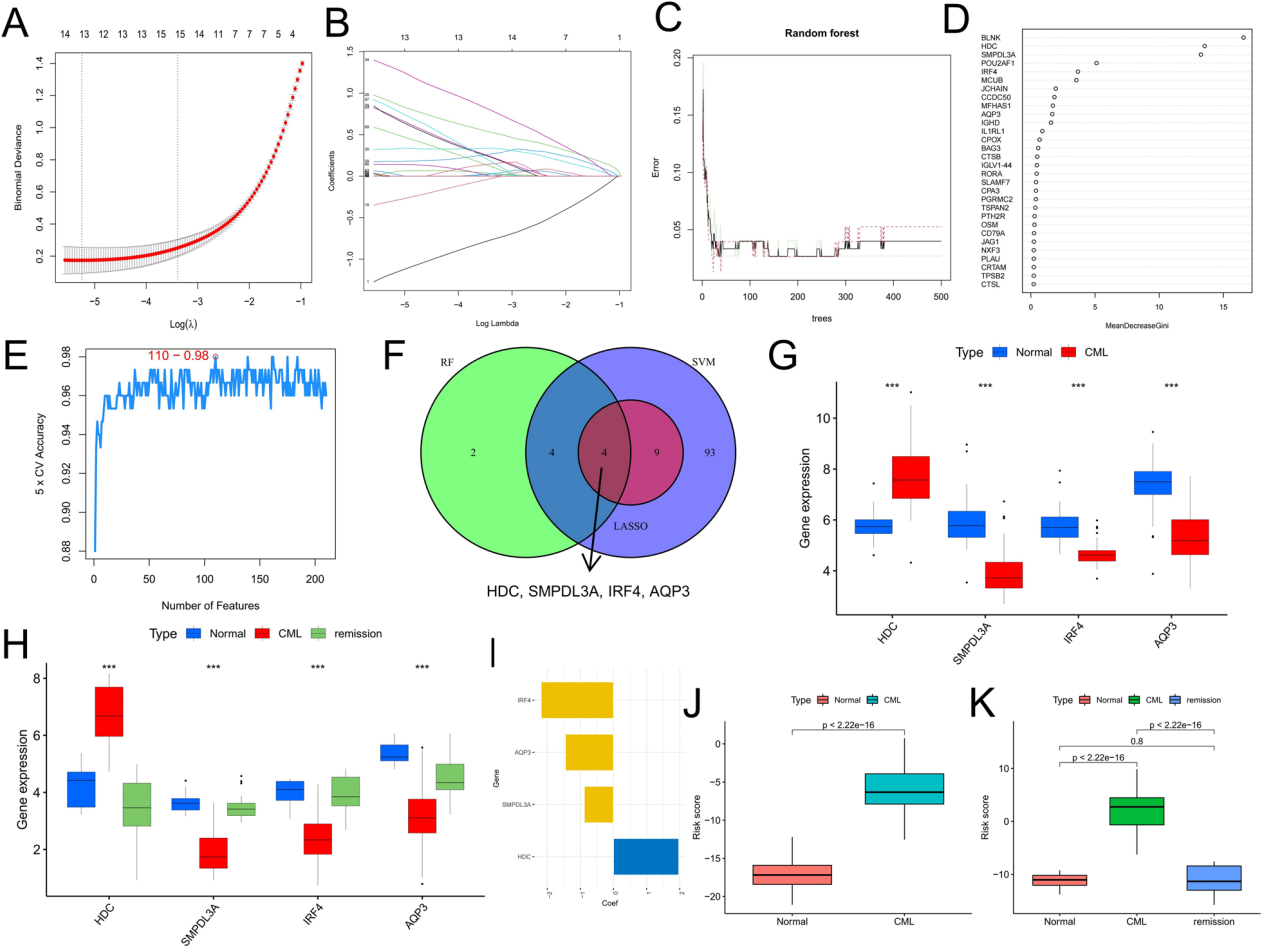
Screening diagnostic markers for CML. **A**, **B** Diagnostic markers were screened by the LASSO regression algorithm. **C**, **D** Diagnostic markers were screened by the RF algorithm. **E** Diagnostic markers were screened by the SVM-RFE algorithm. **F** Venn diagram of variables screened by LASSO, RF and SVM-RFE algorithms. **G** Differences in expression of the four diagnostic genes in the GSE13159 cohort. **H** Differences in expression of the four diagnostic genes in the GSE144119 cohort. **I** Coefficients for the four genes in the risk score model. **J** Distribution of risk scores in the GSE13159 cohort. **K** Distribution of risk scores in the GSE144119 cohort

To further explore the diagnostic value of DEGs in CML. We used LASSO, RF and SVM-RFE algorithms to identify 13, 30 and 110 feature variables related to CML from DEGs, respectively (Fig. 4A–E). Finally, 4 overlapping diagnosis-related genes (HDC, SMPDL3A, IRF4 and AQP3) were selected from the three algorithms. Compared with the normal group, the expression of HDC was significantly up-regulated in CML samples, while SMPDL3A, IRF4 and AQP3 were significantly down-regulated (Fig. 4G). In the validation cohort GSE144119, we observed consistent expression differences; interestingly, four diagnostic genes showed a reversion to normal expression levels in remission patients, which also indicates the value of therapeutic evaluation of these biomarkers (Fig. 4H).

**Fig. 5:**
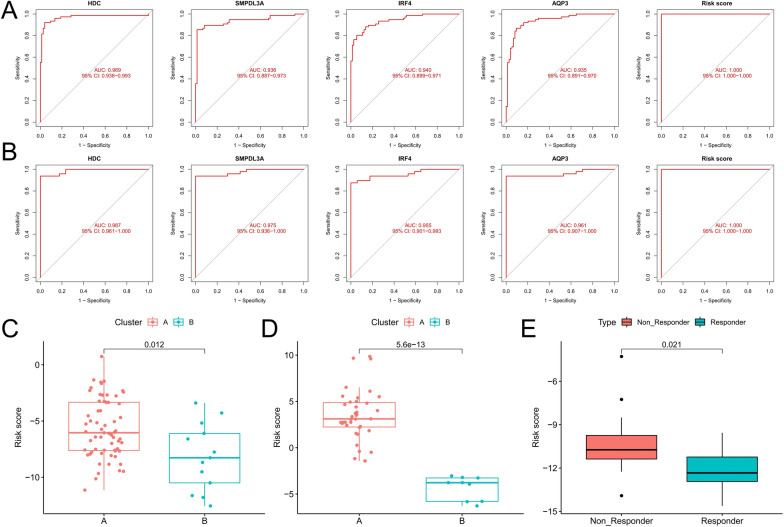
Analysis of the diagnostic value of diagnostic markers. **A**, **B** ROC curve analysis of the diagnostic value of the four diagnostic genes and the risk score in the GSE13159 (**A**) and GSE144119 (**B**) cohorts. **C**, **D** Differences in risk scores between different molecular subtypes in the GSE13159 (**C**) and GSE144119 (**D**) cohorts. **E** Differences in risk scores between patients who responded and did not respond to imatinib treatment in the GSE2535 cohort

Next, we used LASSO regression analysis to construct a risk score model based on four diagnostic genes to explore the diagnostic value of combining these biomarkers. Figure 4I shows the model coefficients for the diagnostic genes (Additional file 1: Table S1). Patients with CML had significantly higher risk scores than those in the normal group (Fig. 4J-K), and the risk score of the remission patients was reduced to the level of the normal group (Fig. 4K). By ROC curve analysis, we confirmed the high diagnostic efficiency of four diagnostic genes in both CML cohorts, while the risk score model further improved the diagnostic power (Fig. 5A, B). In addition, Cluster A had significantly higher risk scores than Cluster B, which observed consistent distributive features in both analysis cohorts (Fig. 5C, D). Meanwhile, in the GSE2535 cohort, 12 patients who did not respond to imatinib had significantly higher risk scores than 16 patients who responded to treatment (Fig. 5E). These results indicate that risk score can not only be used for the diagnosis of CML, but also for the evaluation of molecular subtypes and the prediction of drug resistance.

### Correlation analysis of diagnostic biomarkers with biological characteristics

**Fig. 6:**
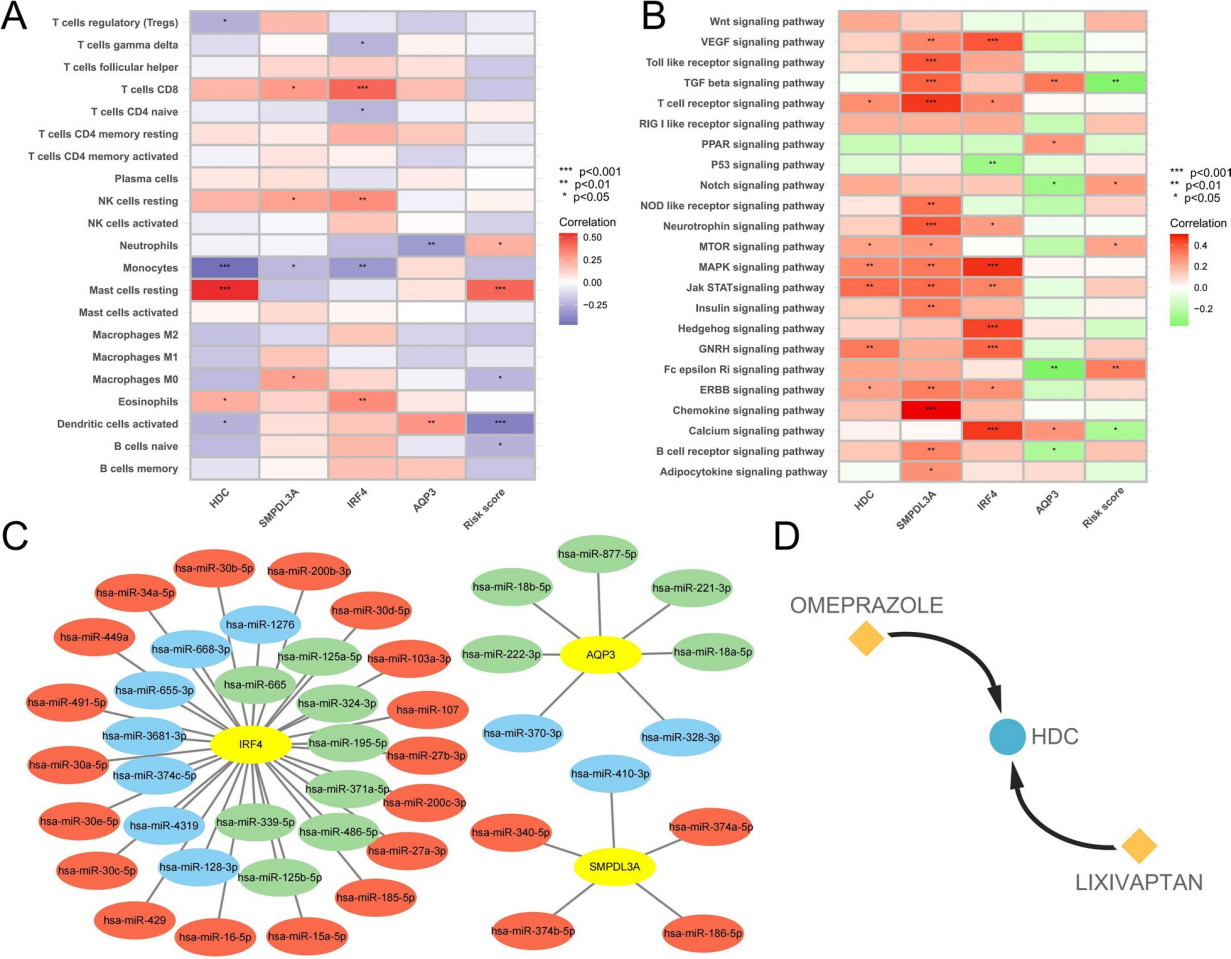
Correlation analysis of diagnostic biomarkers with biological characteristics. **A** Correlation analysis of four diagnostic genes, risk score and immune cells. **B** Correlation analysis of four diagnostic genes, risk score, and cancer-related signaling pathways. **C** Regulatory network of miRNAs and four diagnostic genes; red indicates miRNA expression is up-regulated in CML samples, green indicates expression is down-regulated, and blue indicates that expression data for the relevant miRNAs were not obtained. **D** The interaction network indicates drugs that may have regulatory relationships with HDC

To explore the relationship between these diagnostic biomarkers and biological characteristics of CML, we analyzed the correlation between their expression and the level of immune cell infiltration and the activity of cancer-related signaling pathways in CML samples, respectively. Among them, IRF4 and SMPDL3A were positively correlated with CD8 + T cells and resting NK cells, but negatively correlated with monocytes; HDC was positively correlated with resting mast cells and negatively correlated with monocytes (Fig. 6A). In addition, there was a stronger correlation between diagnostic markers and cancer-related signaling pathways. The higher the expression of SMPDL3A, and IRF4, the stronger the activity of signaling pathways such as VEGF, Toll like receptor, T cell receptor, NOD like receptor, MAPK, JAK-STAT, chemokine, and B cell receptor (Fig. 6B). However, AQP3 shows the opposite correlation.

The above results provide clues to explore the relationship between these biomarkers and the biological characteristics of CML. Meanwhile, considering that most of the diagnostic genes were significantly down-regulated in CML samples, we wondered whether miRNA in CML cells might be involved in the regulation. We first obtained miRNAs with binding sites to diagnostic genes in ENCORI Database, and three diagnostic genes (AQP3, SMPDL3A and IRF4) were retrieved. Subsequently, we obtained the differentially expressed miRNAs between CML samples and normal samples from the GSE90773 dataset. Based on these data, we performed the construction of miRNA regulatory network (Fig. 6C). Many studies have confirmed the regulatory relationship between many miRNA-targets in the network. For example, miR-877 inhibits progression of gastric cancer by down-regulating AQP3 [17], and miR-125b induces bone marrow and B-cell leukemia by inhibiting IRF4 [18], indicating that many up-regulated miRNAs in CML samples may inhibit the expression of target genes. Moreover, HDC is the only diagnostic gene that is upregulated. We predicted drugs interacting with HDC in DGIdb database (https://www.dgidb.org/) to improve clues for related studies, in which omeprazole and lixivaptan may have regulatory effects on HDC (Fig. 6D).

### Validation of clinical independent cohort

**Fig. 7:**
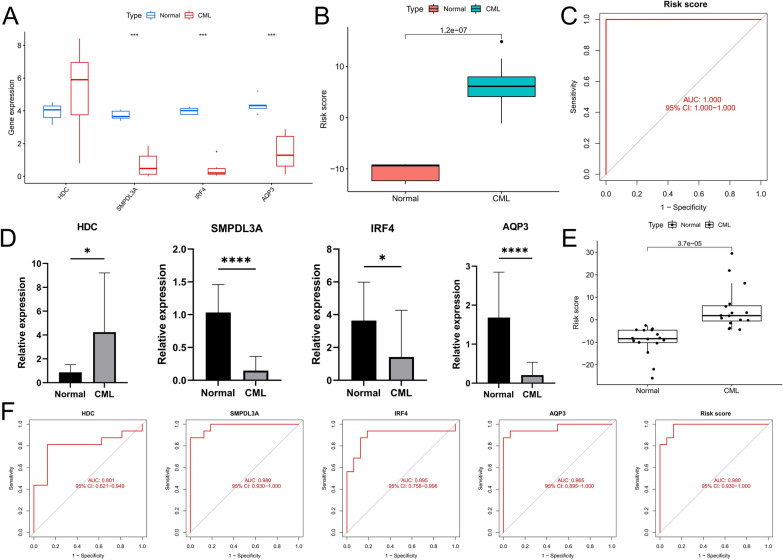
Clinical independent cohort validated diagnostic markers of CML. **A**–**C** Our sequencing cohort validates the differences in four diagnostic genes and risk scores between CML and normal samples and the diagnostic value of risk score in CML. **D**–**F** Our clinical PCR cohort validated the differences in four diagnostic genes and risk scores between CML samples and normal samples, as well as the diagnostic value

To validate the bioinformatics results, we performed validation in our own sequencing data. The results showed upregulated expression of HDC, significantly downregulated expression of SMPDL3A, IRF4, AQP3, and significantly higher risk scores in CML samples compared to normal samples (Fig. 7A, B), and ROC curve analysis also confirmed the high diagnostic efficiency of the risk score (Fig. 7C), which were all consistent with the analysis of GSE13159 and GSE144119 cohorts. In addition, we also performed PCR validation in clinical samples. Encouragingly, the expression characteristics of the four diagnostic genes and the risk score were consistent with the sequencing data, and the ROC curve also confirmed their high diagnostic value (Fig. 7D–F). This indicates that the results of the data analysis are reliable and of potential research value.

### Risk score accurately differentiates CML from other hematological malignancies

**Fig. 8:**
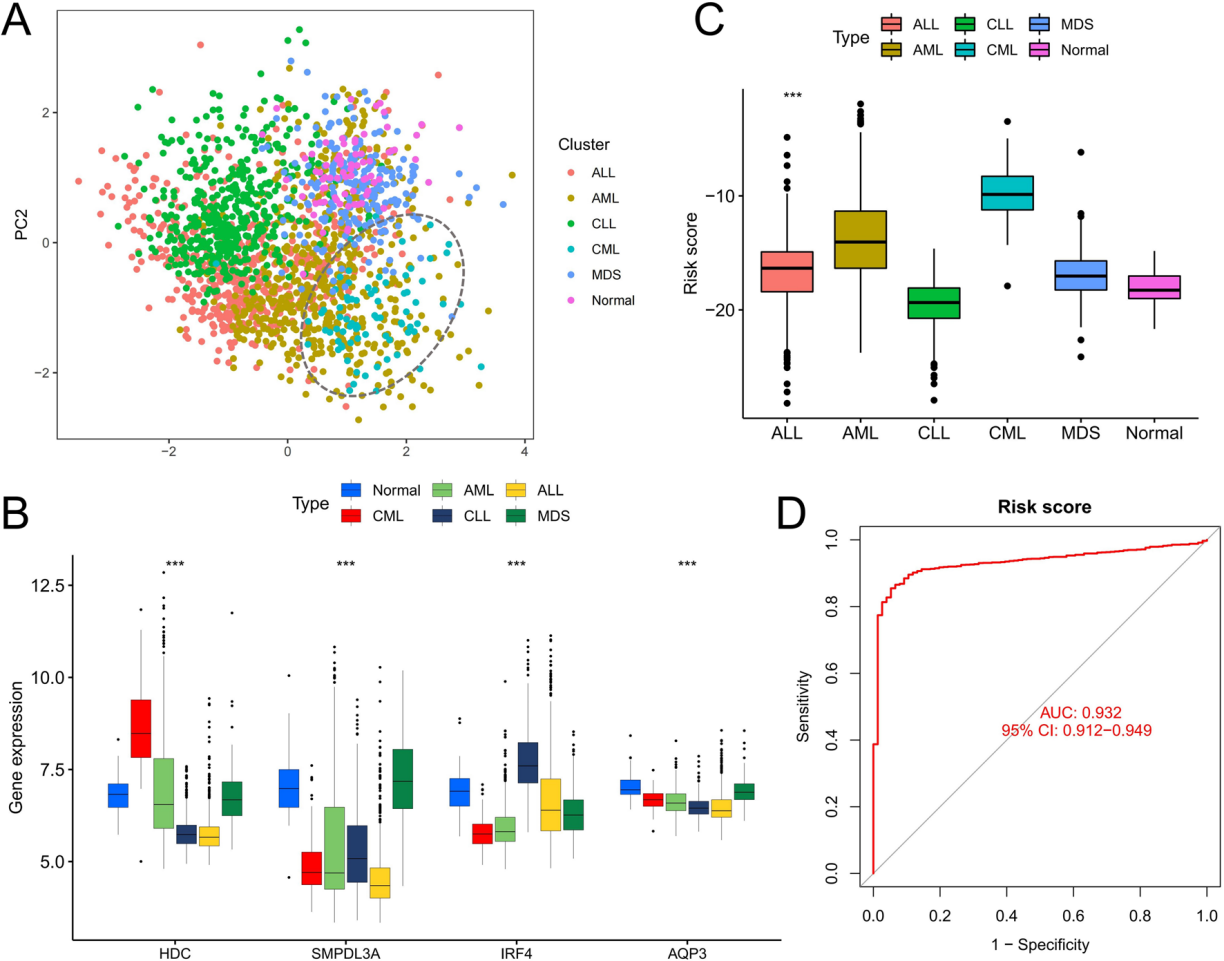
The value of diagnostic markers in the differential diagnosis between CML and other hematological malignancies. **A** PCA plot shows clustering features of CML, AML, CLL, ALL, MDS and normal samples based on expression of the four diagnostic genes. **B** Differential expression of four diagnostic genes in CML, AML, CLL, ALL, MDS and normal samples. **C** Differences in the distribution of risk scores among CML, AML, CLL, ALL, MDS and normal samples. **D** ROC curve analysis of the differential diagnostic value of the risk score in CML versus other hematological malignancies

The GSE13159 cohort contained samples from 750 patients with ALL, 542 patients with AML, 448 patients with CLL, and 206 patients with MDS, which were further used in the differential diagnosis of CML. PCA analysis was based on the expression of four diagnostic genes for clustering. The results showed that CML patients could be clearly distinguished from other patients except some AML patients overlapped (Fig. 8A). Compared with the other five groups of people, the expression level of HDC was the highest in CML patients, while the expression of other diagnostic genes did not show significant differences (Fig. 8B). In addition, patients with CML had the highest risk score (Fig. 8C), and ROC curve analysis showed that risk score could accurately distinguish CML from other hematological malignancies (Fig. 8D).

## Discussion

CML, as a typical hematological tumor induced by chromosomal structural aberrations, the development of TKIs has greatly improved the survival of CML patients and made the cure of the disease possible through long-term drug administration [2]. However, due to individual differences, the emergence of TKI resistance in some patients will lead to accelerated phase and blast phase of the disease, resulting in malignant lesions similar to acute leukemia, which seriously endangers the lives of patients [19]. Moreover, most patients with CML do not have specific symptoms at diagnosis and are often detected during testing for other purposes. At present, the diagnosis of CML is mainly based on an increased number of immature leukocytes in peripheral blood and the identification of the Ph chromosome, as well as the qualitative detection of BCR-ABL1 gene by PCR [20]. In addition, bone marrow aspiration is helpful for further staging of CML patients [21]. However, there are also some patients who do not have a typical Ph chromosome or cannot detect the transcripts of the BCR-ABL1 gene [22]. Therefore, it is important to explore new biomarkers for CML diagnosis and therapeutic evaluation.

In this study, we analyzed the differences in gene expression and TME in CML patients. We found that compared with normal samples, the number of up-regulated and down-regulated genes in CML samples was unbalanced, down-regulated genes were more, and most of them were related to immune-related signaling pathways, indicating that CML samples may be in a state of immunosuppression. In addition, we observed that the activity of fatty acid metabolism-related gene sets such as alpha-linolenic acid metabolism and arachidonic acid metabolism was higher in CML samples. In the bone marrow microenvironment, leukemia cells overexpressed the adipocyte transporter CD36, fully absorbed fatty acids released by adipocytes, and metabolized fatty acids to obtain more energy [23], indicating that CML cells may promote their own survival and proliferation through metabolic reprogramming. We further found that the infiltration of CD8 + T cells was significantly reduced in CML samples, which means that the immune system could not produce enough killing to CML cells, and the high expression of immune checkpoints PD-1, PD-L1, and CTLA4 in CML samples promoted the immune escape of CML cells. In conclusion, we believe that immunosuppression and abnormal energy metabolism may be important reasons for the occurrence and development of CML.

The classification of disease subtypes facilitates a better evaluation of individual patient characteristics to select more targeted treatment options [24]. Here, we identified two distinct molecular subtypes (Cluster A and Cluster B) in CML patients. Cluster A is an immunosuppressive phenotype, showing reduced infiltration of anti-tumor immune cells such as CD8 + T cells, CD4 + T cells and NK cells, and increased activity of energy metabolic pathways such as ROS pathway, glycolytic and mTORC1 signaling pathways. Studies have shown that BCR-ABL1-induced ROS production can be involved in CML progression and TKI resistance [25–27]. Imatinib treatment resulted in reduced glucose uptake in sensitive CML cells, which correlated with inhibition of cell proliferation and induction of apoptosis; however, imatinib-resistant CML cells maintain a highly glycolytic metabolic phenotype [28, 29]. Our prediction results of drug sensitivity also confirmed that Cluster A was less sensitive to imatinib than Cluster B. In addition, BCR-ABL1 directly upregulated the PI3K/AKT/mTOR pathway and led to the induction of glycolysis in CML cells [30], which then promoted cell proliferation, consistent with the significantly increased activity of mTORC1 signaling in Cluster A. The other molecular subtype, Cluster B, exhibited an immune activation phenotype, including significant infiltration of immune cells and activation of immune-related signaling pathways, which suggested that the immune function of patients may play a positive anti-tumor role. However, this molecular subtype also has some immune escape, and we predict that it is more likely to respond to immunotherapy with immune checkpoint inhibitors such as anti-PD-1 and anti-CTLA4, and the drug prediction results also suggest that it also shows higher sensitivity to multiple TKIs.

Finally, we explored the diagnostic biomarkers of CML in depth and identified four diagnostic genes through multiple machine learning methods. Their high diagnostic value was verified in multiple cohorts, and the risk score model constructed by lasso regression analysis further improved the diagnostic efficiency, with significantly higher risk scores in CML patients than in normal subjects. Among them, oncoprotein BCR-ABL1 promoted HDC expression and histamine synthesis in CML cells [31]. Hypermethylation of the CpG motif in the promoter region leads to down-regulation of IRF4 expression [32]. Moreover, high expression of SMPDL3A is associated with poor prognosis in patients with hepatocellular carcinoma, and its high expression promotes the growth of hepatocellular carcinoma [33]. Loss of the water channel AQP3 can enhance the tolerance of CML cells to arsenic trioxide [34], and high expression of AQP3 promotes the development of several cancers such as breast and gastric cancer [17, 35]. The regulatory mechanisms of aberrant expression of HDC and IRF4 in CML were confirmed by relevant experiments, while other genes need to be explored by further research. We performed sequencing and PCR validation on clinical samples, respectively, and the expression characteristics of four diagnostic genes and risk score were consistent with the results of bioinformatics analysis, indicating the accuracy of data analysis and the feasibility of clinical application. In addition, the risk score can also effectively identify CML patients with different molecular subtypes or different responses to imatinb treatment, and the risk scores of Cluster A patients and imatinb-resistant patients were significantly higher. We also evaluated the value of the risk score in the differential diagnosis of CML, and the risk score was highest in CML compared with other hematological tumors. Due to fluctuations in the expression of individual genes or incidental detection errors, risk score as a comprehensive indicator can greatly reduce the impact of detection errors. These results reveal the robustness of the diagnostic genes and the constructed risk score model in the diagnosis of CML, and the risk score can be used as a single indicator to effectively evaluate the treatment response and molecular subtype of CML. At present, the therapeutic evaluation of CML is mainly to detect the expression level of BCR-ABL1 fusion gene [20]. However, due to the existence of the independent resistance mechanisms, which will give more advantages to the pathological growth of CML cells [36], the detection based on the BCR-ABL1 fusion gene may not be able to accurately evaluate the treatment response and develop more personalized treatment plans. As an auxiliary indicator, the risk score model we constructed has potential clinical application value and is beneficial for identifying the immune subtypes of CML patients to play an evaluative role in immunotherapy response. miRNAs may play an important regulatory role in the expression of these genes, and our constructed miRNA regulatory network provides a certain clue.

In conclusion, this project revealed differences in biological features such as gene expression, signaling pathway activity and immune cell infiltration between CML patients and normal subjects based on transcriptomic sequencing data. The identified molecular subtypes help to better evaluate the disease status of patients and select personalized treatment regimens. Biomarkers identified and validated based on multiple machine learning approaches have high diagnostic value, which also complements the diagnostic approach to CML. However, our research also has some limitations. The size of clinical samples in the validation cohorts is small, and it is a single center sample. The application of diagnostic signatures to clinical applications still requires more sample validation. In addition, due to the diversity of detection methods for gene expression, such as RNA-seq, gene chip and RT-qPCR, the gene expression levels detected by each method are different. Therefore, further normalized data are needed to determine the risk score threshold of a specific situation, which requires a large amount of sample data. The accurate threshold value will be beneficial to accurately judge the disease status of patients and make personalized treatment plan. We will expand the sample size and use multi-center samples to verify the application value of risk score in subsequent studies, and explore the biological functions and regulatory mechanisms of diagnostic genes in CML through more basic experiments.

## Supplementary Information

**Additional file 1: Figure S1**. Identification of molecular subtypes of CML in the GSE144119 cohort. Differences in expression of DEGs (A), infiltration of 22 immune cells (B) between the two molecular subtypes.

**Additional file 2. Table S1**. Model genes and “Coef” value.

## Data Availability

All data used in this work can be acquired from the Gene-Expression Omnibus (GEO; https://www.ncbi.nlm.nih.gov/geo/) database.

## References

[1] Nash I (1999). Chronic myeloid leukemia. N Engl J Med.

[2] Jabbour E, Kantarjian H (2018). Chronic myeloid leukemia: 2018 update on diagnosis, therapy and monitoring. Am J Hematol.

[3] Lugo TG, Pendergast AM, Muller AJ, Witte ON (1990). Tyrosine kinase activity and transformation potency of bcr-abl oncogene products. Science.

[4] Hochhaus A (2017). Long-Term outcomes of imatinib treatment for chronic myeloid leukemia. N Engl J Med.

[5] Iqbal Z (2013). Sensitive detection of pre-existing BCR-ABL kinase domain mutations in CD34+ cells of newly diagnosed chronic-phase chronic myeloid leukemia patients is associated with imatinib resistance: implications in the post-imatinib era. PLoS ONE.

[6] Deininger MW, O'Brien SG, Ford JM, Druker BJ (2003). Practical management of patients with chronic myeloid leukemia receiving imatinib. J Clin Oncol.

[7] Hong M (2020). RNA sequencing: new technologies and applications in cancer research. J Hematol Oncol.

[8] Qi J (2023). Combined scRNAseq and Bulk RNAseq analysis to reveal the dual roles of oxidative stress-related genes in acute myeloid leukemia. Oxid Med Cell Longev.

[9] Hue SS (2023). Tissue-Specific microRNA expression profiling to derive novel biomarkers for the diagnosis and subtyping of small B-Cell lymphomas. Cancers.

[10] Joshi A, Sasumana J, Ray NM, Kaushik V. in *Advances in Bioinformatics* (eds Vijai Singh & Ajay Kumar) 351–364 (Springer Singapore, 2021).

[11] Branford S, Shanmuganathan N (2019). NGS in CML—new standard diagnostic procedure?. HemaSphere.

[12] Némethová V, Rázga F (2017). Overexpression of ABCB1 as prediction marker for CML: how close we are to translation into clinics?. Leukemia.

[13] Park JH (2019). HMGCLL1 is a predictive biomarker for deep molecular response to imatinib therapy in chronic myeloid leukemia. Leukemia.

[14] Fu J (2020). Large-scale public data reuse to model immunotherapy response and resistance. Genome Med.

[15] Jiang P (2018). Signatures of T cell dysfunction and exclusion predict cancer immunotherapy response. Nat Med.

[16] Subhomoi B, Amit J, Vikas K, Anupam Nath J. in Drug Development Life Cycle (eds Akhtar Juber, Badruddeen, Ahmad Mohammad, & Khan Mohammad Irfan) Ch. 6 (IntechOpen, 2022).

[17] Zhu H, Wu Y, Kang M, Zhang B (2020). MiR-877 suppresses gastric cancer progression by downregulating AQP3. J Int Med Res.

[18] So AY (2014). Dual mechanisms by which miR-125b represses IRF4 to induce myeloid and B-cell leukemias. Blood.

[19] Poudel G, Tolland MG, Hughes TP, Pagani IS (2022). Mechanisms of resistance and implications for treatment strategies in chronic myeloid leukaemia. Cancers.

[20] Jabbour E, Kantarjian H (2022). Chronic myeloid leukemia: 2022 update on diagnosis, therapy, and monitoring. Am J Hematol.

[21] Orazi A, Neiman RS, Cualing H, Heerema NA, John K (1994). CD34 immunostaining of bone marrow biopsy specimens is a reliable way to classify the phases of chronic myeloid leukemia. Am J Clin Pathol.

[22] Costello R, Sainty D, Lafage-Pochitaloff M, Gabert J (1997). Clinical and biological aspects of Philadelphia-negative/BCR-negative chronic myeloid leukemia. Leuk Lymphoma.

[23] Tabe Y (2017). Bone marrow adipocytes facilitate fatty acid oxidation activating AMPK and a transcriptional network supporting survival of acute monocytic leukemia cells. Cancer Res.

[24] Sunil Krishnan G, Joshi A, Kaushik V. in Advances in Bioinformatics (eds Vijai Singh & Ajay Kumar) 303–315 (Springer Singapore, 2021).

[25] Antoszewska-Smith J, Pawlowska E, Blasiak J (2017). Reactive oxygen species in BCR-ABL1-expressing cells—relevance to chronic myeloid leukemia. Acta Biochim Pol.

[26] Głowacki S (2021). Relationship between oxidative stress and imatinib resistance in model chronic myeloid leukemia cells. Biomolecules.

[27] Sánchez-Sánchez B (2014). NADPH oxidases as therapeutic targets in chronic myelogenous leukemia. Clin Cancer Res.

[28] Kominsky DJ (2009). Abnormalities in glucose uptake and metabolism in imatinib-resistant human BCR-ABL-positive cells. Clin Cancer Res.

[29] Klawitter J (2009). Metabolic characteristics of imatinib resistance in chronic myeloid leukaemia cells. Br J Pharmacol.

[30] Kim JH (2005). Activation of the PI3K/mTOR pathway by BCR-ABL contributes to increased production of reactive oxygen species. Blood.

[31] Aichberger KJ (2006). The CML-related oncoprotein BCR/ABL induces expression of histidine decarboxylase (HDC) and the synthesis of histamine in leukemic cells. Blood.

[32] Ortmann CA (2005). Down-regulation of interferon regulatory factor 4 gene expression in leukemic cells due to hypermethylation of CpG motifs in the promoter region. Nucleic Acids Res.

[33] Zhang Y (2022). Sphingomyelin phodiesterase acid-like 3A promotes hepatocellular carcinoma growth through the enhancer of rudimentary homolog. Front Oncol.

[34] Sobh A (2019). Functional profiling identifies determinants of arsenic trioxide cellular toxicity. Toxicol Sci.

[35] Milković L, Čipak Gašparović A (2021). AQP3 and AQP5-potential regulators of redox status in breast cancer. Molecules.

[36] Osman AEG, Deininger MW (2021). Chronic Myeloid Leukemia: modern therapies, current challenges and future directions. Blood Rev.

