# Supplementary material for: Molecular subtypes predict therapeutic responses and identifying and validating diagnostic signatures based on machine learning in chronic myeloid leukemia
Source: Cancer Cell Int. 2023 Apr 6;23:61. doi: 10.1186/s12935-023-02905-x (PMC10080819; doi:10.1186/s12935-023-02905-x)
Supplement: Supplementary file 1 — Additional file 1: Figure S1. Identification of molecular subtypes of CML in the GSE144119 cohort. Differences in expression of DEGs (A), infiltration of 22 immune cells (B) between the two molecular subtypes. [file 12935_2023_2905_MOESM1_ESM.docx]

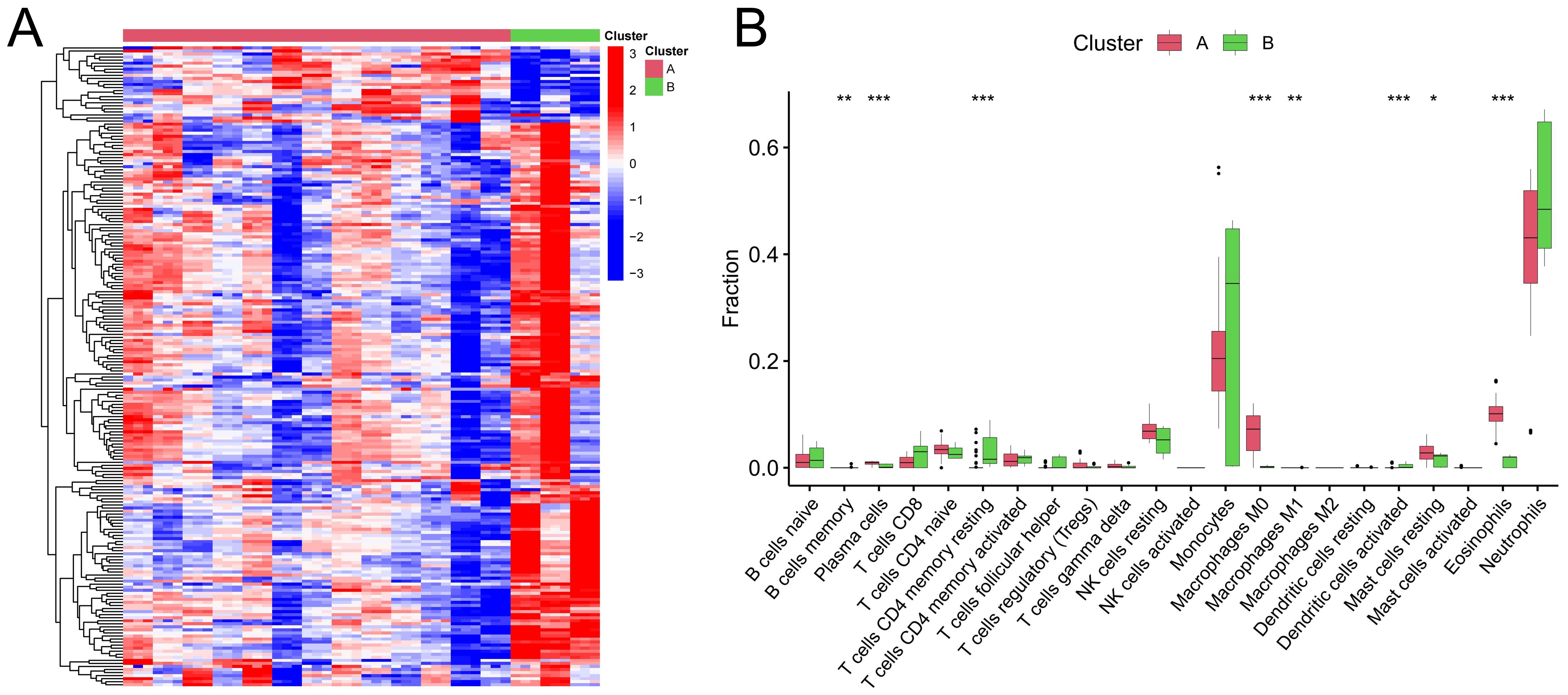


**Figure S1. Identification of molecular subtypes of CML in the GSE144119 cohort.** Differences in expression of DEGs (A), infiltration of 22 immune cells (B) between the two molecular subtypes.
